# Probing the effects of streptomycin on *Brassica napus* germination and assessing its molecular interactions using extensive molecular dynamics (MD) simulations

**DOI:** 10.1038/s41598-023-46100-4

**Published:** 2023-11-04

**Authors:** Rohit Patel, Karan Prajapati, Dweipayan Goswami, Meenu Saraf

**Affiliations:** Department of Microbiology and Biotechnology, University School of Sciences, Gujarat University, Ahmedabad, Gujarat 380009 India

**Keywords:** High-throughput screening, Virtual drug screening, Plant molecular biology, Plant physiology

## Abstract

Antibiotics are chemical compounds that are used to treat and prevent disease in humans and animals. They have been used in animal feed for over 60 years and are widely used in industrial farming. Antibiotics can have negative environmental impacts, including the potential to contribute to the development of antibiotic-resistant organisms. They can enter the environment through various pathways, including the manufacturing process, the direct application of antibiotic-laden manure to fields, and through grazing animals. Antibiotics that are given to animals can be excreted from where they can enter soil and groundwater which enable their entry in plants. Streptomycin is an antibiotic that is used against a range of gram-positive and gram-negative bacteria, but its use has led to the development of antibiotic resistance in some pathogens. It has also been shown to have negative impacts on a range of plant species, including tobacco, tomato, and wheat. Although, the major effect of streptomycin on plant physiology have been studied, the molecular mechanisms at play are barely understood in plant body. In current study, we examined the impact of streptomycin on germination of *Brassica napus* and then using docking, MM-GBBSA and MD simulations identified key proteins that interact with streptomycin by performing rigorous computational screening of 106 different proteins. Our finding suggest that streptomycin might be interacting with acyl-CoA oxidases, protochlorophyllide reductase B and leucoanthocyanidin dioxygenase based on simulation and docking analysis.

## Introduction

Antibiotics have been used in animal feed for over 60 years and are a global trend due to the reliance on industrial farming to produce food. There are over 150 antibiotics from either natural source such as bacteria, fungi, or are semisynthetic modifications of natural compounds or are entirely synthetic. The annual world-wide use of antibiotics, including those used in veterinary and medical settings, is estimated to be between 100,000 and 200,000 tons^1–3^. Veterinary antibiotics (VA's) are utilized to treat, prevent, and control disease in animals and for growth promotion. There are over 2000 veterinary pharmaceutical products derived using 400 active chemical components to treat different species of animals. The environmental impact of VA's, including their potential to contribute to the development of antibiotic-resistant organisms, has been well studied. Antibiotics used in livestock enter the environment through various pathways, including pharmaceutical companies, the drug manufacturing process, the direct application of antibiotic-laden manure to fields, and through grazing animals. These antibiotic residues can be found in sewage, activated sludge, digested sludge, and urban biosolids^3,4^. In organic agricultural practices, the direct application of animal manure is the primary source of antibiotic entry into the environment. These antibiotics can then find their way into soil, the aquatic ecosystem, and plants. When animals are given antibiotics, they are excreted either as the parent compound or as metabolites^3^. The rate of excretion can vary from 40 to 90% for different antibiotics. Some studies have shown that as much as 90% of some antibiotics may be excreted as the parent compound, while others estimate that 25% of the oral dose of tetracycline is excreted in faeces and 50–60% as the parent compound or active metabolite in urine^5^. Farm soil and groundwater serve as two primary reservoirs of residual antibiotics. From this stage antibiotics can enter plant ecosystems and exhibit their negative impacts on plants^3,6,7^.

Streptomycin (PDB chemical id: SRY), is an antibiotic of first-generation aminoglycoside class, isolated from bacterium *Streptomyces griseus*. Structurally, it consists of three rings (1) streptidine (2) streptose and (3) *N*-methyl-l-glucosamine. Here, streptidine ring is a variant of scyllo-inositol with guanidino group substitutions at positions of first and third hydroxyl groups. This ring is bound to streptose ring which is having linkage with *N*-methyl-l-glucosamine. Initially, SRY was effective against broad range of gram-positive and gram-negative bacteria as it targets the protein synthesis machinery through interactions with 16S rRNA sub-segment of 30S prokaryotic ribosome subunit^8,9^. Among prokaryotes this antibiotic has been successfully used against pathogens such as *Francisella tularensis*, *Yersinia pestis*, *Mycobacterium tuberculosis*, and so on. Currently, indiscriminate use of antibiotic has initiated an antibiotic resistance among several of these pathogens^10^. As a resistance mechanisms bacteria have developed enzymes such as aminoglycoside-2''-phosphotransferase-IIa and aminoglycoside nucleotidyltransferases. These enzymes identify the aminoglycoside drugs and deactivates them though the cofactor-dependent modification of the amino and hydroxyl groups to protect the bacteria^11,12^.

Whereas for eukaryotes effects of streptomycin are reported on tobacco, broad bean, tomato, radish, soybeans, and wheat. SRY was determined to be toxic for seedlings of tomato and radish at concentrations above 50 ppm. Furthermore, radish exposed to concentrations higher than 3000 ppm were showing leaf yellowing due to chlorosis. Among other species, *Euglena gracilis* var. *bacillar* were subjected to 100 ppm of streptomycin which resulted in the permanent bleaching of organism^13^. Similar bleaching effects for barley, rye, carrot tumor tissue, pine seedlings, chlorella, and cress has been reported^14,15^. Wheat showed no physical growth retardation up to 200 ppm^15^. Here, depending on plants the inhibitory concentrations of SRY were found to be varying with wide range of effects from outright toxicity, lateral root development inhibition, reduced plant length, and chlorosis. Among most common effects observed was absence of chlorophyll from green plants and protista. Another study that explored the effects of streptomycin on plant flowering showed that streptomycin promoted flowering, and streptomycin is also believed to be inhibiting protein synthesis in chloroplasts^16^. Furthermore, actively growing plants that are exposed to light along with SRY shows permanent removal of chlorophyll whereas if plants are incubated in dark conditions with SRY, removal of SRY is reinstating the chlorophyll. The growth of *A. thaliana* can be inhibited through exposure to SRY with effects such as inhibition of expansion of leaves, development of stem internodes, coleoptiles, and roots. In such conditions *Arabidopsis* shows no enhancement of growth even when sugar is supplied to the medium^17,18^.

Apart from these inhibitory effects, report of streptomycin toxicity prevention by manganese is also available^14^. Moreover, the antagonistic effects of gibberellic acid on streptomycin promoted growth inhibition are also available in the literature. Such effect gave birth to the hypothesis that streptomycin might be hindering formation and availability of gibberellic acid in plant bodies^19^. Alongside, it was also suggested that SRY might be forming a chelating complex with manganese, making them unavailable to the plants. Additive, studies on relation of SRY and Mn have provided the evidence of further correlation^15^. Addition of bacterial gene responsible for aminoglycoside resistance, streptomycin phosphotransferase, makes plants resistant to SRY based inhibitions which has been explored extensively in molecular biology for developing GMO plants^20^. Currently the molecular mechanisms of SRY interaction in plant bodies are poorly understood. One of the molecular targets, has been the eukaryotic ribosome but the exact mechanisms involved for eukaryotes are still an illusion^21^. Although we have plenty of information on physiological changes promoted by SRY, the exact molecular mechanisms are not explored sufficiently.

**Figure 1:**
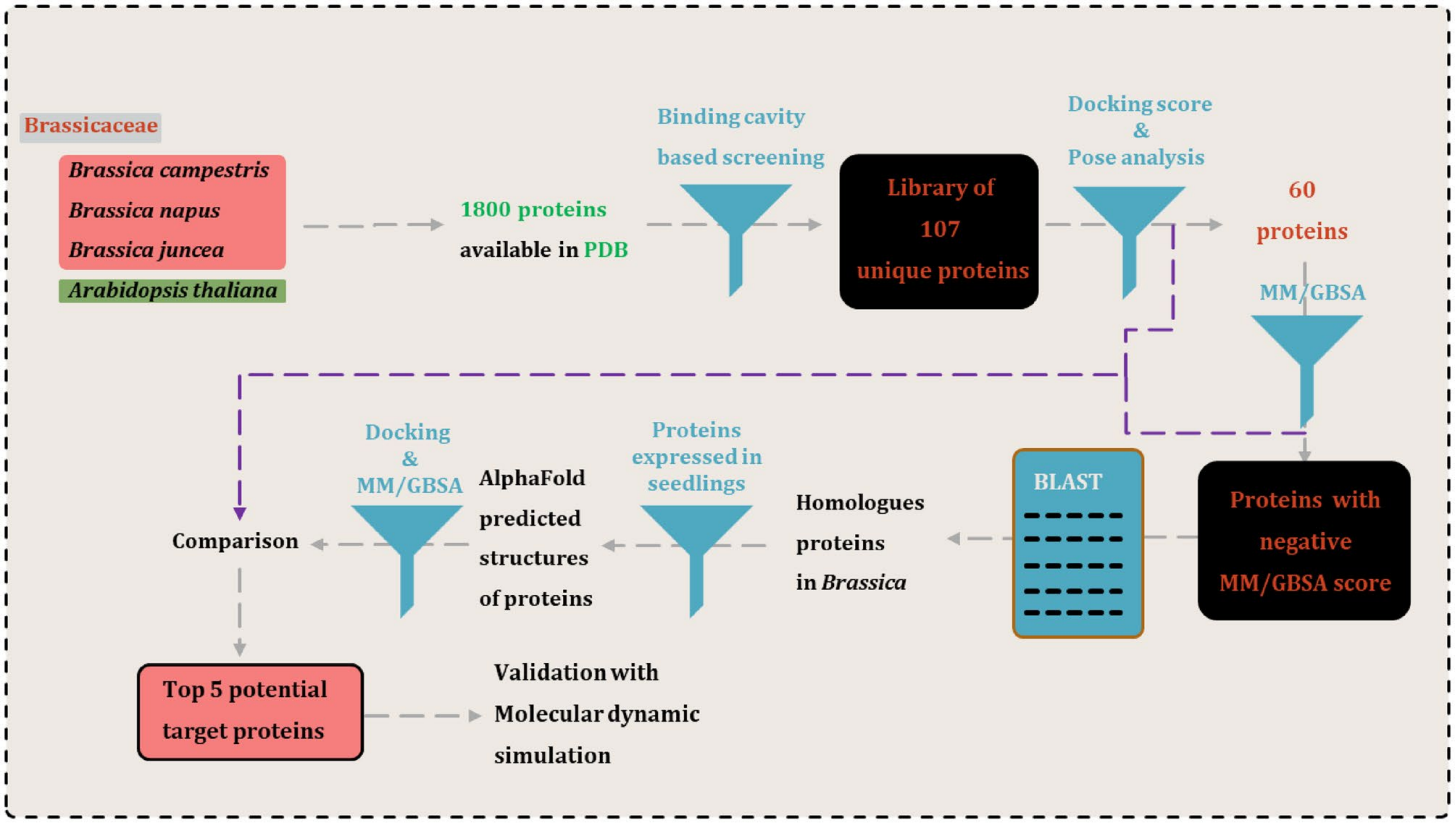
Workflow of the study to screen the potential protein targets for streptomycin.

In current study, computational methods such as molecular docking, MM-GBSA, and molecular dynamic simulation were explored to identify the potential targets of SRY in young seedling of *Brassica napus* (Fig. 1). These tools have been extensively explored for development of novel drugs and their interactions with proteins available in eukaryotic systems such as mosquito^22^, human^23^, fungi^24^ etc. Here, similar methodologies are being explored for plant bodies. In study, three targets have been identified in the young seedling that are having high probability of being streptomycin targets.

## Material and methods

### Seed germination assay

The effect of streptomycin on seed germination was assayed using *Brassica napus* seeds (variety: NANDI-321; NANDI SEEDS PVT. LTD). During experiment utilization of any growth medium was avoided as effect on germination and initial growth were the target of the study. Large agar plate with 150 ml of agar containing 300 µg/mL streptomycin was prepared after solidification 100 seeds of *B. napus* were added in the plate. After 72 h of incubation, 100 seeds were taken out and differences in the total length of germinated seeds were measured. For control group of the study 100 seeds of *B. napus* were incubated for 72 h in agar plate without streptomycin. To increase the statistical accuracy of the data entire experiment was performed in triplicate. Furthermore, the seeds length and germination data obtained from the assay was subjected to statistical *T* test to validate if the changes were significant. *T* test was carried out using the Excel-365. Data analysis tool available in data panel was used to access the *t* test: Two samples assuming unequal variance option and *T* test was performed at alpha value of 0.05.

### Identification of potential targets

Data from protein data bank (PDB; https://www.rcsb.org/) 3D structure repository was explored to identify the potential targets for streptomycin in plant body. PDB currently has 28 unique proteins associated with name *Brassica*. Here, 28 proteins are very limited number of proteins. To avail a broad range of protein targets, we also used proteins from *Arabidopsis thaliana* in the study. This organism is a commonly studied host and is a member of the *Brassicaceae* family, alongside *Brassica*. Additionally, studies have reported that the growth of *Arabidopsis* is also hindered by streptomycin. Using the filter mechanisms of PDB website unique proteins that harbours the ligands with at least three rings in their structure were identified and they were utilized for further docking and molecular simulation study.

### Preparation of proteins

Once the proteins with co-crystallized 3 ringed ligands were identified they were retrieved from the PDB and minimized for molecular docking using ‘protein preparation wizard’ of Schrodinger maestro (Schrödinger Release 2021-2). Here, proteins were imported using their PDB ID. Once imported, using ‘Preprocess’ feature bond orders were assigned, along with addition of hydrogen. Furthermore, proteins were optimized further for docking by incorporating features such as the formation of disulfide bonds, conversion of selenomethionines to methionines, and filling in missing side chains and loops, all of which are available in the 'import and process' panel. Then, features from the ‘review and modify’ panel were applied to remove irrelevant water molecules, ligands, and chains of protein. Finally, in the ‘refine’ panel protein was optimized using H-bond assignment feature and subsequently minimized using OPLS4 forcefield^25^.

### Preparation of ligands

In current study every protein was docked with two types of ligands, (1) co-crystallized ligands and (2) streptomycin ligand. Co-crystallized ligands were obtained from the PDB entry itself using the split function available in maestro. Whereas structure of streptomycin was obtained from the PubChem (CID 19649)^26^. Once obtained all the ligands were prepared using ‘LigPrep’ wizard of maestro. Here, all the ligands were neutralized and prepared using OPLS4 forcefield^5,27^. Once minimized ligands were used for molecular docking^28,29^.

### Receptor grid generation

Minimized protein files were used as an input file for generating the grids to identify the location where ligand docking is supposed to take place. ‘Receptor grid generation’ wizard of maestro was used for this process where ligand of interest was selected and grid size for each ligand was set using ‘site’ panel. Furthermore, in site panel ‘advanced settings’ option was employed to restrict the docking area to binding cavity where size for each ligand was selected separately. Also, ‘constraints’ panel was employed to specify the metal ions whenever they were present in the binding cavity of protein to further increase the reliability of docking.

### Protein–ligand docking and MMGBSA

Previously minimized and prepared protein–ligand files and receptor grid files were used for docking analysis using ‘ligand docking’ wizard of maestro. Here, location of receptor gid file was specified in the wizard whereas in the ‘ligands’ panel, location of minimized ligands was specified rest of the parameters were kept default in this panel. In ‘settings’ panel of ‘ligand docking’ wizard, precision was set to XP (extra precision) and ligand sampling was kept flexible whereas rest the parameters were kept default. In the ‘constrains’ panel previously specified metal ions whenever present were selected as part of docking using ‘grid-based’ constraints option. Finally, for each docking top 5 poses were analysed, and their docking scores were noted. Furthermore, previously minimized co-crystallized ligands were redocked and once the integrity of grid was validated with pose observation and interactions of redocked ligand, the protein was employed for docking of streptomycin. In case when redocked ligands were not producing correct poses grids were considered faulty and reprepared for more accurate results. Protein–ligand docking score were noted and whenever score for any protein-streptomycin complex was at par or better than protein-redocked ligand (co-crystallized ligand) complex was subjected to MMGBSA for further selection of protein.

Calculation to get endpoint ∆DG was performed using semiquantitative methods, molecular mechanics generalized born surface area (MMGBSA)^30^. Here, minimized docked complex of ligand–receptor generated during docking were used (Schrödinger Release 2021-2). OPLS2005 force field was applied to the docked complex to determine the binding energy for each receptor–ligand complex. Following equation was employed for free energy calculations:

$$\Delta {\text{GBind }} = \, \Delta {\text{EMM }} + \, \Delta {\text{GSolv }} + \, \Delta {\text{GSA}}$$

Here ΔEMM represents the variation between the minimized energy of the receptor–ligand complexes; ΔGSolv represents the variation between the GBSA solvation energy of the receptor–ligand complexes and the sum of the solvation energies for the protein and ligand. ΔGSA contains sum of the surface area energies in the protein and ligand and the difference in the surface area energies for the complexes.

### Homolog identification using BLAST

Once the aforementioned filtration pipeline was applied it produced the names of ideal protein targets for streptomycin available within *Brassica* spp. and *Arabidopsis thaliana*. Here, the target organism of this study is *Brassica* hence, the identification of homologs of targets identified in *Arabidopsis thaliana* was next major step. To achieve this target BLAST tool available on UNIPROT (https://www.uniprot.org/blast) was used to perform BLAST sequence alignment analysis. For performing the BLAST analysis UniProtKB reference proteomes + Swiss-Prot was used as a target database. The taxonomy of search was restricted to *Brassica* species and rest of the parameters were kept as default. The most suitable sequences were determined based on two parameters namely the percent coverage and the genus of *Brassica*. Since the study was focused on field *Brassica*, *Brassica campestris*, *Brassica juncea*, and *Brassica napus* were prioritized over others. Once the homologs were identified the structures submitted by alphafold were downloaded and studied for their reliability using SAVES v6.0 (https://saves.mbi.ucla.edu/) and used for docking and MD analysis. Apart from the structural assessment, chimera was used to determine the similarities in secondary structure features of protein using match-maker option available in tools. Along with similarity search the coordinates for binding site in homolog protein were also determined here and used in subsequent docking studies.

Redocking assays were performed using methods described ahead and MMGBSA assessment was carried out for both the ligands that are originally occupying the binding cavity and streptomycin.

### Molecular dynamic simulation

Molecular dynamic (MD) simulation was performed to verify the stability of complex formed during docking analysis. To perform the MD simulation Desmond (Schrödinger Release 2021-4) released by D.E. Shaw research was used^31^. Here, the validity and precision of each MD were ensured by taking protein-redocked ligand as a control. To perform the simulation docked poses of protein were exported in pdb format and imported in the Desmond. Once imported ‘system builder’ wizard was used to create a system for simulation where water was employed as solvent system with ‘TIP3P’ option. System was built in orthorhombic box of 10 Å × 10 Å × 10 Å dimension. Once the system was ready ‘molecular dynamics’ wizard was used to specify the parameters of simulation.

### Ethical approval

Experimental research on seeds/plants, including the collection of seed/plant material, complied with relevant institutional, national and international guidelines and legislation. Current study does not involve research that require any additional ethical approval as any endangered plant species are not part of this study

## Results

### Seed germination assay

**Figure 2:**
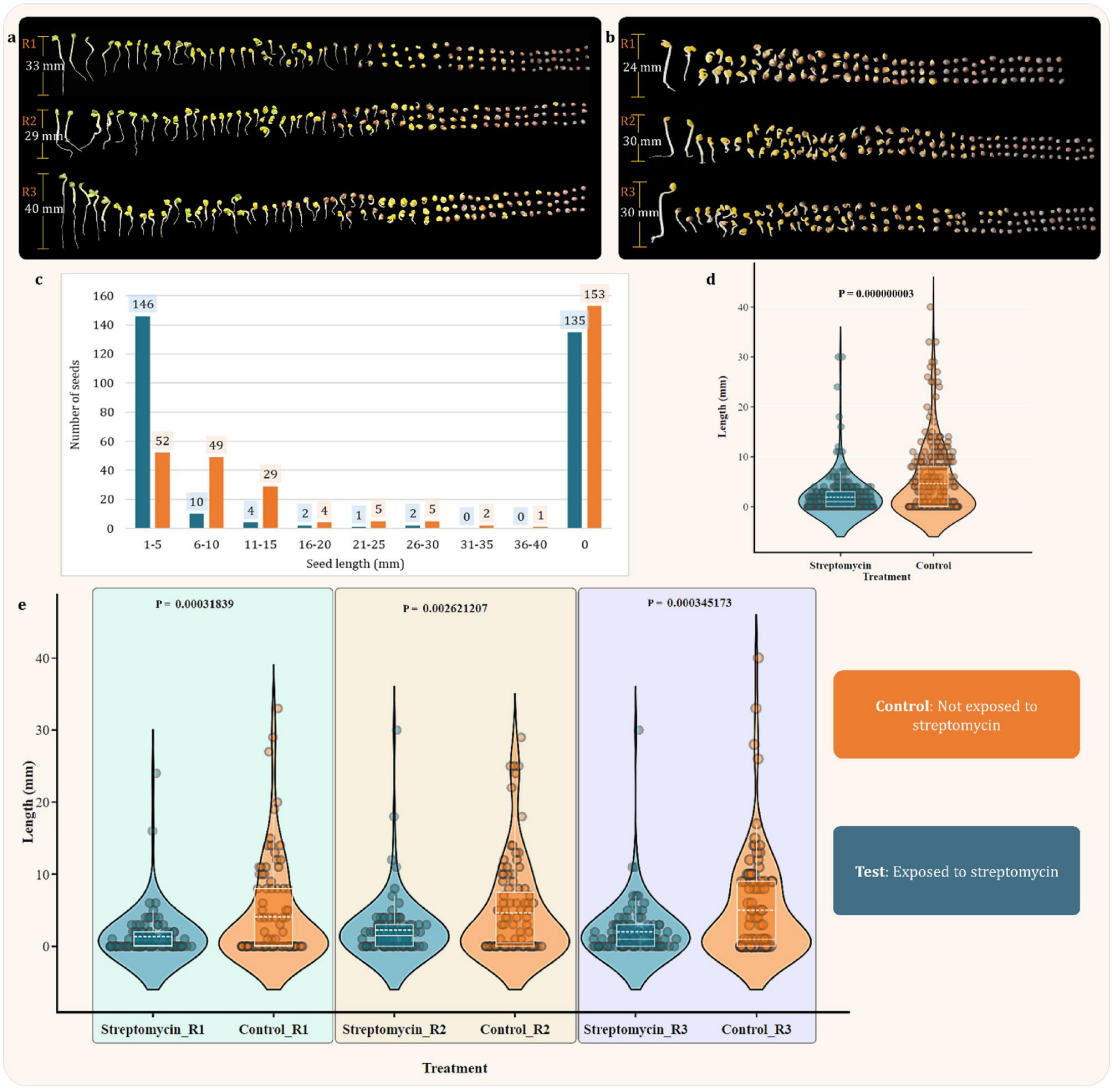
Seed germination assay: (**a**) each row contains 100 seeds of *B. napus* without exposure to streptomycin (**b**) each row contains 100 seeds of *B. napus* exposed to streptomycin (**c**) represents the trend changes observed in the length of germinated seeds after exposure to streptomycin for 72 h. Here, all the number of seeds from all the three trials of control and test are combined and groups are formed for length measurement of 5 mm. (**d**) Violin plot represents the distribution of combined seed length in streptomycin and control group where, the data from all the 300 seeds from triplicate is merged and *T* test *p* value for these data is found to be 0.000000003. (**e**) Here, the violin plots of all three trials R1, R2 and R3 are separately presented with *p* values produced by *T* test for each pair (All the violin plots are produced using plotly online tool (https://chart-studio.plotly.com)).

Effect of streptomycin on germination of *B. napus* seeds was estimated using agar seed germination assay method. Here, seeds were allowed to germinate in the presence (test) and absence of streptomycin (Control) for 72 h. Changes in number of seeds germinated and attained length were recorded after 72 h and simple bar plot was used to represent the quantitative information along with photographs of each trial to get the estimate of qualitative effect. As represented in Fig. 2a (control) in all 3 trial seeds attained maximum hights of 33 mm, 29 mm, and 40 mm respectively. Whereas for test (Fig. 2b) the maximum length of germinated seed was noted to be 24 mm, 30 mm and 30 mm which shows reduction compared to control. When these results are combined into groups (Fig. 2c) according to their lengths it is apparent that number of seeds with length less than 5 mm is higher in population subjected to streptomycin.

Furthermore, the impact of streptomycin on the seed length of *B. napus* across three independent trials, was studied using the *T* test. *T* tests results were statistically significant in all trials. The lengths of all seeds across three trials were summed up and data was combined in to two groups streptomycin and control to create a violin plot (Fig. 2d). Moreover, this same data was then utilized to conduct *T* test and *P* value was found to be 0.00000003 (Fig. 2d). Apart from this, the *T* test was also carried out on for all three trials individually. In Trial 1 (R1), the *p* value was 0.00031839, with average seed lengths of 1.34 for the streptomycin group and 4.08 for the control. In Trial 2 (R2), the *p* value was 0.002621207, with mean seed lengths of 2.26 and 4.66 for the streptomycin and control groups, respectively. Finally, in Trial 3 (R3), the *p* value was 0.000345173, and the mean seed lengths were 2.01 for the streptomycin group and 5.01 for the control (Fig. 2e). In all trials, the control groups not only had significantly greater mean seed lengths but also exhibited higher variance compared to the streptomycin groups. These consistent results across multiple trials establish that streptomycin has a statistically significant and inhibitory effect on *B. napus* seed length. The uniformity of these findings strengthens their reliability and raises questions about the biological mechanisms through which streptomycin affects plant growth. Therefore, the study serves as compelling evidence that streptomycin exposure has a detrimental impact on the seed length of *B. napus.*

### Identification of streptomycin target proteins

Data from protein data bank (PDB) 3D structure repository was explored to identify the potential targets for streptomycin in *Arabidopsis thaliana*. Repository is currently having ~ 1800 different proteins structures of the *Arabidopsis thaliana*. Data of these different structures was obtained from PDB, and to further identify the potential targets various bound ligands within these proteins were analysed. As the structure of streptomycin is having 3 ringed structures, only proteins bound to ligand structures with 3 or more rings were used. Among 509 different unique ligands bound with different proteins of *Arabidopsis,* 154 ligands were identified to have a structure that has 3 or more rings. Proteins associated with these ligands were identified and repeated entries were removed which resulted in identification of 107 unique proteins that were capable of harbouring ligands with 3 or more rings in their structure. These proteins were used for further bioinformatics-based studies.

### Molecular docking and MMGBSA

In the current study, molecular docking and MMGBSA both were used as a filter to further narrow down the search of ideal target for streptomycin. As molecular docking alone is not an ideal filter for such study it was employed as a primary filter to get the proteins for MMGBSA which produces more reliable results. MMGBSA assessment is performed to check the spontaneity of any protein–ligand interaction where more negative the value of ΔGBind higher the chances of random interaction occurrence upon collision in the cytosol. Molecular docking was applied on 107 different proteins and following aspects were observed to narrow down the search (1) streptomycin’s ability to produce pose with the protein (2) difference between docking score of streptomycin and native ligand crystallized with protein. In cases where streptomycin was not forming any pose with proteins they were removed from the further consideration. Also, whenever it was observed that the docking score of streptomycin is not at par with the docking score of co-crystallized ligand of protein they were also filtered out from any further consideration. Using this approach 47 different proteins were eliminated and rest of the 60 proteins were subsequently subjected to MMGBSA assessment. After MMGBSA assessment protein–ligand complex that produced the negative binding energy were selected for further assessment. Among these selected targets, proteins that were actively expressed during the seed germination and seedling or young developmental stages were identified and their homologs in *Brassica* species were recognized in UNIPROTKB using BLAST tool (Table 1).

**Table 1:** Target homologs identified in *Brassica campestris* using BLAST analysis.

Entry ID	Name of protein	Homolog_in_*Brassica*	Identity (%)	E-value
2IX5	Acyl-CoA oxidase	A0A397Y570	89.1	0
7JK9	Protochlorophyllide reductase B	A0A398AMM1	94.5	0
1RP0	Thiamine thiazole synthase,	A0A397XQM3	92.3	0
2BRT	Leucoanthocyanidin dioxygenase	A0A398AN85	91	0
3P86	Serine/threonine-protein kinase CTR1	A0A397XTZ2	90.5	0

### Homolog identification using BLAST

In search of homologs BLAST results provided the hits for *Brassica campestris, Brassica rapa subsp. Pekinensis, Brassica oleracea var. oleracea*, etc. Here, the hits for *Brassica campestris* were taken into consideration for further studies as it is field mustard closest relative of *Brassica napus*, plant in question. Among these search results, *Brassica napus* sequence were desirable but there were none to be found that were giving satisfactory identification and E-values. Table 1 represents the homolog sequences that were finalized for further studies along with their percent identification and E-values. 3D structure superimposition and sequence similarity assay results are given in figures. Furthermore, this homolog proteins were also subjected to molecular docking and pose arrangements were studied.

### Homolog docking and MM-GBSA

In docking and MM-GBSA assessment five different protein–ligand complexes for each target protein were considered. Here, for the assessment of grid and docking protocol accuracy co-crystallized ligand was redocked in the same cavity and the pose arrangement and scores were compared with that of protein–ligand complexes from PDB. Once the accuracy of grid was confirmed streptomycin was docked in the cavity. Similarly, the binding cavities of homolog proteins were identified and both native ligand and streptomycin were docked in the cavity. Scores generated from MMGBSA for all the five different complexes for each protein are given in Table 2. Along with the total energy change of the system ΔGBind, Coulomb energy (ΔGCoulomb), Hydrogen-bonding correction (ΔGHbond), Lipophilic energy (ΔGLipo), pi–pi packing correction (ΔGPacking) and Van der Waals energy (ΔGvdW) for each complex are also given in the table. Here, ΔG bind values were the key values for deciding which protein–ligand complex are ideal and further simulation analysis were conducted. At this stage A0A398AN85, A0A397ZBK1, A0A398AJT5 and A0A397ZR47 did produced the good scores with co-crystallized ligand but failed to produce negative scores with streptomycin in the assessment hence, they were removed from any further consideration.

**Table 2:** MMGBSA energy scores of protein targets and their homologs.

Sr. no.	Protein	Ligand ^&^	ΔG_Bind_	ΔG_Coulomb_	ΔG_Hbond_	ΔG_Lipo_	ΔG_Packing_	ΔG_vdW_
1	**2IX5**^**@**^	**FAD**^**#**^	− 103.1	− 25.2	− 7.3	− 23.1	− 5.8	− 64.5
		**FAD**^**##**^	− 112.64	− 60.51	− 5.53	− 25.85	− 5.91	− 70.10
		**SRY**	− 67.44	− 16.77	− 4.56	− 28.88	0	− 43.87
	A0A397Y570*	**FAD**^**##**^	− 89.75	− 5.23	− 4.80	− 28.29	− 10.08	− 82.68
		**SRY**	− 64.26	− 33.29	− 4.30	− 28.79	0	− 46.57
2	**7JK9**^**@**^	**NDP**^**#**^	− 92.70	− 175.44	− 15.11	− 16.50	− 2.83	− 98.07
		**NDP**^**##**^	− 67.36	− 49.84	− 9.81	− 12.05	− 2.01	− 61.61
		**SRY**	− 56.64	− 45.72	− 3.07	− 7.85	0	− 30.60
	A0A398AMM1*	**NDP**^**##**^	− 61.10	− 39.80	− 6.68	− 9.19	− 0.001	− 60.10
		**SRY**	− 43.81	− 34.35	− 6.11	− 9.08	0	− 48.49
3	**1RP0**^**@**^	**AHZ**^**#**^	− 80.83	30.98	− 9.85	− 14.24	− 3.16	− 81.09
		**AHZ**^**##**^	− 106.17	− 68.96	− 7.23	− 15.57	− 2.60	− 72.84
		**SRY**	− 53.86	− 55.67	− 7.031	− 6.85	0	− 30.74
	A0A397XQM3*	**AHZ**^**##**^	− 88.58	− 49.71	− 5.33	− 13.56	− 2.60	− 64.86
		**SRY**	− 46.32	− 47.40	− 4.97	− 9.33	0	− 46.62
4	**3P86**^**@**^	**STU**^**#**^	− 70.21	39.43	− 1.62	− 20.39	− 0.46	− 57.84
		**STU**^**##**^	− 71.67	− 17.78	− 1.60	− 20.59	− 0.58	− 60.59
		**SRY**	− 45.28	− 24.16	− 3.02	− 19.86	0	− 45.88
	A0A397XTZ2*	**STU**^**##**^	− 47.66	− 14.31	− 1.31	− 18.96	− 0.09	− 49.88
		**SRY**	− 41.91	− 43.25	− 4.30	− 11.66	0	− 43.00
5	**2BRT**^**@**^	**NAR**^**#**^	− 37.70	− 15.12	− 1.67	− 10.82	− 4.38	− 33.15
		**NAR**^**##**^	− 35.29	− 11.17	− 1.71	− 10.85	− 4.36	− 33.11
		**SRY**	− 51.74	− 76.00	− 5.98	− 13.86	0	− 35.40
	A0A398AN85*	**NAR**^**##**^	− 39.94	− 20.72	− 2.83	− 9.56	− 3.60	− 27.27
		**SRY**	− 7.03	− 16.08	− 5.29	− 10.74	0	− 40.96

@ = PDB ID; * = UNIPROT ID; & = PDB chemical ID; # = scores generated from PDB protein–ligand complex; ## = scores generated from redocked protein–ligand complex.

### MD simulations

#### UniProt ID A0A397Y570

**Figure 3:**
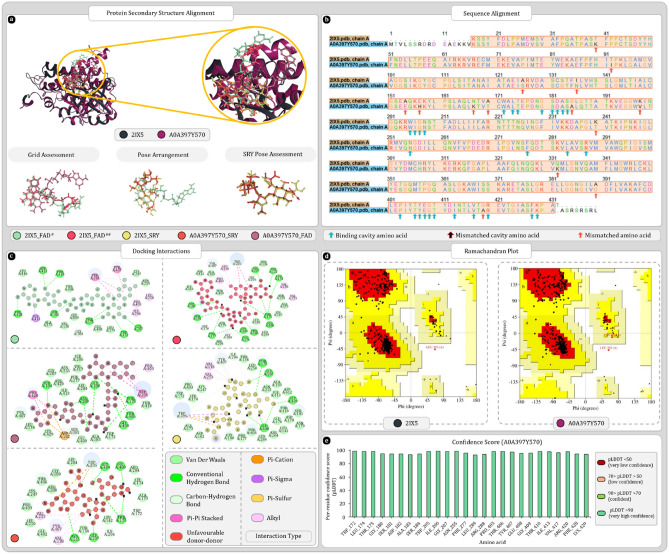
Secondary structure, sequence alignment, and docking analysis of 2IX5 and A0A397Y570 with binding cavity analysis of A0A397Y570 (**a**) 3D superimposition of 2IX5 and A0A397Y570 along with 3D arrangement of ligands: Grid assessment (PDB pose of FAD in 2IX5, redocked pose of FAD in 2IX5, docked pose of FAD in A0A397Y570); Pose arrangement (PDB pose of FAD in 2IX5, docked pose of SRY in 2IX5, docked pose of SRY in A0A397Y570); SRY Pose arrangement (SRY in binding cavity of A0A397Y570 and 2IX5) (**b**)** s**equence alignment and identification of mismatched amino acids in binding cavity of A0A397Y570 (**c**) docking interactions of SRY and FAD with 2IX5 and A0A397Y570 (**d**) ramachandran plot of 2IX5 and A0A397Y570 (**e**) per residue scores produced by AlphaFold for each residue of binding cavity.

Uniport ID A0A397Y570 was found to be the most ideal match for the 2IX5. Results of secondary structure superimposition are given in Fig. 3a. Furthermore, sequence alignment reveals no mismatched binding cavity amino acid between two amino acid sequences Fig. 3b. Among 411 compared amino acid 14 mismatches were found but none of these amino acids changed the secondary structure components of A0A397Y570. Ramachandran plots of both proteins were analysed which showed that 90% of residues of 2IX5 were in most favoured region whereas for A0A397Y570 92.1% residues were in most favoured region (Fig. 3d). Confidence scores produced by AlphaFold were also checked for the amino acids that were forming the binding cavity which reveals the all the important residues were having high confidence score (Fig. 3e). Also, the molecular docking study shows that all the docked ligands were occupying the same binding cavity. The redocked FAD ligand was also properly arranged in the binding cavity and was found to be superimposing the co-crystallized FAD whereas only solvent exposed tail region, adenosine-5’-diphosphate, was differently arranged which is expected as this region of ligand has more space to move around than the isoalloxazine ring located in the depth of binding cavity. Moreover, the pose arrangement study reveals that inside cavity docked streptomycin for both proteins was arranged similarly and was occupying the same region as isoalloxazine ring of FAD (Fig. 3a). Docking scores of 2IX5 with FAD and streptomycin were respectively found to be − 17.09 and − 11.39 kcal/mol whereas for homolog A0A397Y570, FAD and streptomycin were giving docking score of − 12.60 and − 11.57 kcal/mol. Docking interactions produced by 2IX5 and A0A397Y570 are given in Fig. 3c. After docking these best docking complexes were further analysed for MMGBSA where 2IX5-FAD complex from PDB produced − 103.1 kcal/mol. After redocking score fluctuated a bit and was found to be − 112.64 kcal/mol this change is expected as binding cavity was completely cleaned by removing water molecules. Here, homolog protein produced -89.75 kcal/mol MMGBSA score with FAD. Whereas with SRY score of both 2IX5 and homolog was respectively − 67.44 and − 64.24 kcal/mol. After exporting the complexes of homolog with FAD and SRY both were used for 100 ns MD simulation to check the stability of FAD and SRY within the binding cavity of A0A397Y570.

**Figure 4:**
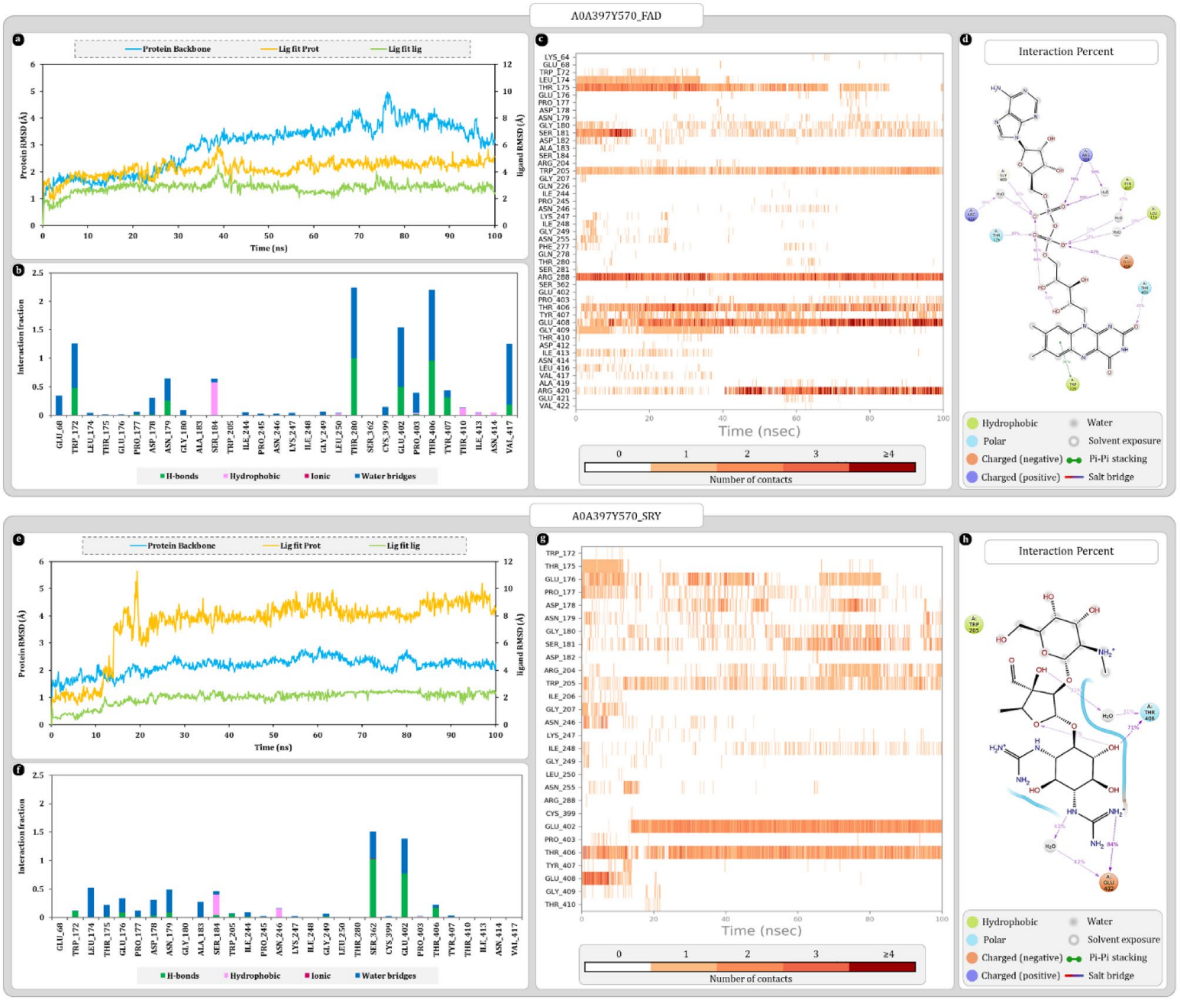
Molecular dynamics simulation trajectory analysis plots (**a**) root mean square deviation (RMSD) calculation of A0A397Y570-FAD complex and (**b**) interaction fraction analysis plot of A0A397Y570 with FAD (**c**) timeline of interactions between A0A397Y570 and FAD (**d**) percent interaction profile of FAD with A0A397Y570 (**e**) root mean square deviation (RMSD) calculation of A0A397Y570-SRY complex (**f**) interaction fraction analysis plot of A0A397Y570 with SRY (**g**) timeline of interactions between A0A397Y570 and SRY (**h**) percent interaction profile of SRY with A0A397Y570.

The RMSD calculations aim to measure the changes in the protein–ligand poses with respect to reference frame which in this case is the docking pose of complex. In the plot left-Y axis represents the RMSD values for protein backbone whereas right-Y axis represents the ligand RMSD. Protein RMSD values for A0A397Y570-FAD complex were stabilized around 3.4 ± 0.5 Å after 30 ns of simulation and remains as such throughout the simulation. On the other hand, ligand RMSD value “Lig Fit Prot” represents the changes occurring in ligand pose with respect to entire complex that were stabilized around 4.5 ± 0.5 Å. “Lig Fit Lig” values use the ligand alone as a reference which were found to be stabilizing around 3.0 ± 0.5 Å. Here, around 38 ns spike in ligand RMSD was noted which was attributed to the movement of adenosine-5′-diphosphate which returns to the stable position within 2 ns which brings the RMSD down again (Fig. 4a). Protein–ligand contact timeline graph shows that most consistently interacting amino acids throughout the interactions are TRP205, ARG288, THR406, and GLU408. Among other amino acids LEU174, THR175, SER181 and GLY409 shows significantly consistent interactions with FAD for initial 38 ns but after slight change in pose of adenosine-5’-diphosphate, these interactions either stop or their frequency reduces. After slight pose change ARG420 interacts more strongly with FAD even making up to 4 contacts (Fig. 4c). Most frequently occurring interactions among amino acids are hydrogen bonds and water bridges. ARG288, THR406, and GLU408 makes the most consistent hydrogen bonds alongside THR175, SER181 and GLY409 also makes hydrogen bond but occurrence is relatively scattered throughout the simulation. An oxygen from α-phosphate of adenosine-5′-diphosphate strongly interacts with ARG288 to form the most stable hydrogen bond along with water bridge. Moreover, another oxygen from the same phosphate forms hydrogen bond and water bridges with GLY409 and ARG420. Another oxygen from β-phosphate of adenosine-5′-diphosphate forms hydrogen bonds and water bridges with GLU408, and THR175. Also, the isoalloxazine ring of FAD forms a hydrophobic interaction with TRP205 along with hydrogen bond and water bridge with THR406 (Fig. 4b–d).

Protein RMSD values for A0A397Y570-SRY complex were stabilizing around 2.3 ± 0.5 Å after 25 ns and remains consistent throughout the simulation. Values of “Lig Fit Prot” for SRY ligand stabilizes around 9.0 ± 0.5 Å which suggests major changes in the position of ligand compared to reference pose. Whereas “Lig Fit Lig” values were stabilizing around 2.2 ± 0.3 Å which suggest no major changes ligand structure. Here, the spike of nearly 7 Å was noted around 13 ns when the SRY that was docked within the cavity moves at the mouth of cavity and stabilizes there (Fig. 4e). *N*-methyl-l-glucosamine ring of streptomycin was docked deep within the cavity that interacted with GLU408 initially but after 13 ns SRY is forced out of the cavity towards the mouth of the cavity and the interaction breaks. Whereas components of streptidine ring strongly and consistently interacted with THR406 and GLU402 via hydrogen bonds and water bridges throughout the interaction (Fig. 4f). Moreover, THR406 also makes water bridge with streptose ring and stabilizes it. THR175, GLU176, PRO177, ASP178, ASN179, GLY180, SER181 and TRP205 combinedly contributed significantly towards stabilizing *N*-methyl-l-glucosamine ring via mostly water bridges except TRP205 which was making hydrophobic pi–pi stacking interaction. Additionally, TRP205 was also interacting with streptose ring (Fig. 4f–h).

#### UniProt ID A0A398AMM1

**Figure 5:**
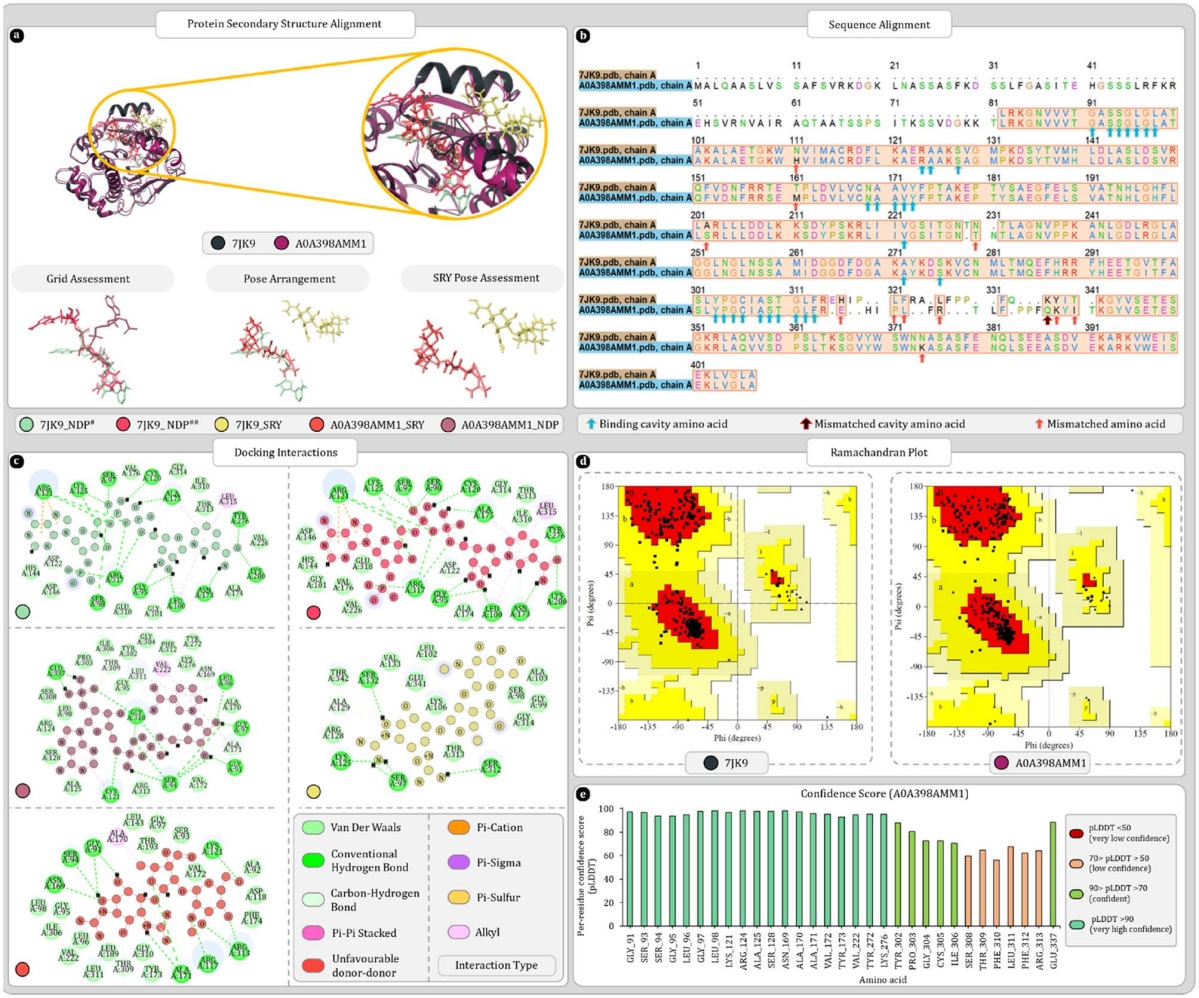
Secondary structure, sequence alignment, and docking analysis of 7JK9 and A0A398AMM1 with binding cavity analysis of A0A398AMM1 (**a**) 3D superimposition of 7JK9 and A0A398AMM1 along with 3D arrangement of ligands: Grid assessment (PDB pose of NDP in 7JK9, redocked pose of NDP in 7JK9, docked pose of NDP in A0A398AMM1); Pose arrangement (PDB pose of NDP in 7JK9, docked pose of SRY in 7JK9, docked pose of SRY in A0A398AMM1); SRY Pose arrangement (SRY in binding cavity of A0A398AMM1 and 7JK9) (**b**)** s**equence alignment and identification of mismatched amino acids in binding cavity of A0A398AMM1 (**c**) docking interactions of SRY and NDP with 7JK9 and A0A398AMM1 (**d**) ramachandran plot of 7JK9 and A0A398AMM1 (**e**) per residue scores produced by AlphaFold for each residue of binding cavity.

Uniport ID A0A398AMM1 was found to be the most ideal match for the 7JK9. Results of secondary structure superimposition are given in Fig. 5a. Furthermore, sequence alignment reveals single amino acid mismatch in the binding cavity at position 336 where 7JK9 is having lysine, A0A398AMM1 has glutamine (Fig. 5b). Throughout the entire sequence number of mismatches were found furthermore, there were two big sequence gaps present. Regardless of these changes the secondary structure arrangement of the homolog protein remains significantly similar to that of 7JK9. Ramachandran plot analysis of 7JK9 reveals that 91.7% of residues were in favoured regions whereas for A0A398AMM1 this number was 92.4% which suggests overall good quality of protein. AlphaFold confidence scores of binding cavity amino acid were in range of either confident or highly confident except for SER308, THR309, GLY310, LEU311, PHE312, and ARG313 with scores in the range of low confidence (Fig. 5e).

Also, the molecular docking study shows that all the docked NDP ligands were occupying the same binding cavity (Fig. 5a). The redocked NDP ligand was arranged slightly differently in the binding cavity, but the orientation of ligand was like co-crystallized NDP. Here, nicotinamide ring was buried deep within the cavity and adenosine was located near the mouth of the cavity where it has more space for movement. Moreover, the pose arrangement study reveals that inside cavity docked streptomycin for both proteins was different, and it was occupying the different regions of cavity (Fig. 5a,c,d). The SRY in 7JK9 is located on the surface of the protein whereas for A0A398AMM1, streptidine ring of SRY is overlapping with that of ribose ring associated with nicotinamide which bound deep within the cavity. Docking scores of 7JK9 with NDP and streptomycin were respectively found to be − 12.481 and − 6.09 kcal/mol whereas for homolog A0A398AMM1, NDP and streptomycin were giving docking score of − 13.18 and − 9.866 kcal/mol. After docking these best docking complexes were further analysed for MMGBSA where 7JK9-NDP complex from PDB produced − 92.70 kcal/mol. After redocking score and was found to be − 67.36 kcal/mol. Here, homolog protein produced − 61.10 kcal/mol MMGBSA score with NDP. Whereas with SRY score of both 7JK9 and homolog was respectively, − 56.64 and − 43.81 kcal/mol.

**Figure 6:**
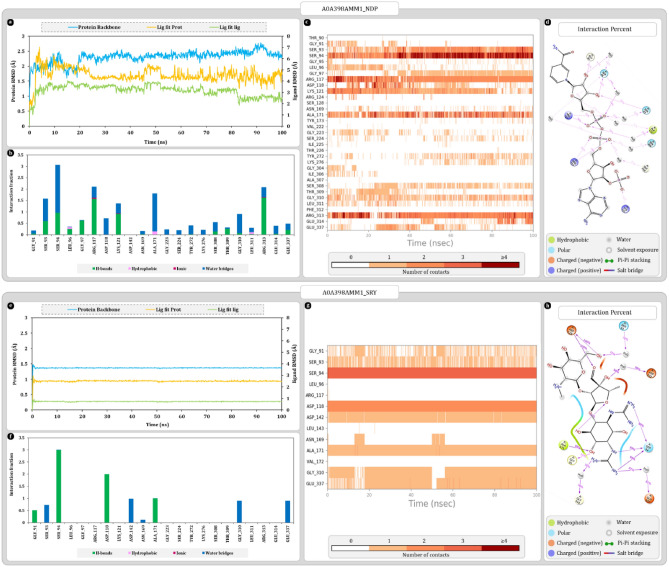
Molecular dynamics simulation trajectory analysis plots (**a**) root mean square deviation (RMSD) calculation of A0A398AMM1-NDP complex and (**b**) interaction fraction analysis plot of A0A398AMM1 with NDP (c) timeline of interactions between A0A398AMM1 and NDP (**d**) percent interaction profile of NDP with A0A398AMM1 (**e**) root mean square deviation (RMSD) calculation of A0A398AMM1-SRY complex (**f**) interaction fraction analysis plot of A0A398AMM1 with SRY (**g**) timeline of interactions between A0A398AMM1 and SRY (**h**) percent interaction profile of SRY with A0A398AMM1.

Protein RMSD values for A0A398AMM1-NDP complex were stabilized around 2.3 ± 0.5 Å and remains as such throughout the simulation. On the other hand, ligand RMSD value “Lig Fit Prot” were stabilized around 4.5 ± 0.5 Å. “Lig Fit Lig” values were found to be stabilizing around 2.5 ± 1.0 Å. Here, most of the ligand RMSD fluctuations were attributed to the movement of adenosine tail of NDP (Fig. 6a). Protein–ligand contact timeline graph shows that most consistently interacting amino acids throughout the simulations were SER93, SER94, ARG117, ALA171, and ARG313. Among these amino acids SER93, SER94, GLY97, THR309, and GLY310 forms the deep end of cavity that contributes to stabilizing nicotinamide and associated ribose of ligand (Fig. 6a–c). Phosphate linker of NDP ligand was most reactive portion of ligand that interacted with ARG117, and ARG313, to make most stable hydrogen bonds throughout the simulation. Besides, amino acids like SER93, SER94, ALA171, SER308, and GLY310 were also making water bridges with phosphate linker. On the deep end of cavity, ribose sugar associated with nicotinamide ring was interacting with SER93, SER94 and GLY97 to form hydrogen bonds and water bridges. Whereas at the mouth of cavity phosphate associated with ribose sugar was interacting with LYS121 to form hydrogen bond (Fig. 6a,d).

Protein RMSD values for A0A398AMM1-SRY complex were stabilized around 1.4 ± 0.2 Å and remains as such throughout the simulation. Among all the complexes simulated this has been the most stable interaction of SRY. On the other hand, ligand RMSD value “Lig Fit Prot” were stabilized around 2.5 ± 0.2 Å. “Lig Fit Lig” values were found to be stabilizing around 0.7 ± 0.2 Å (Fig. 6e). Protein–ligand contact timeline graph shows that most consistently interacting amino acids throughout the simulations were SER94, ASP118, ASP142 and ALA171. Among these amino acids SER94, ASP118, and ALA171 were forming hydrogen bonds whereas ASP142 was participating in water bridge formation. SER94 and ASP118 were respectively forming two and three interactions during the entire 100 ns simulation (Fig. 6f–h). Here, ASP118 was forming hydrogen bonds with *N*-methyl-l-glucosamine and streptose ring of streptomycin. Furthermore, the streptidine ring was being stabilized by SER94, GLY91 and ALA171 with direct hydrogen bond formations. Here, the SER94 was forming 3 hydrogen bonds with streptidine ring being the major contributor in stability (Fig. 6f–h).

#### UniProt ID A0A397XQM3

**Figure 7:**
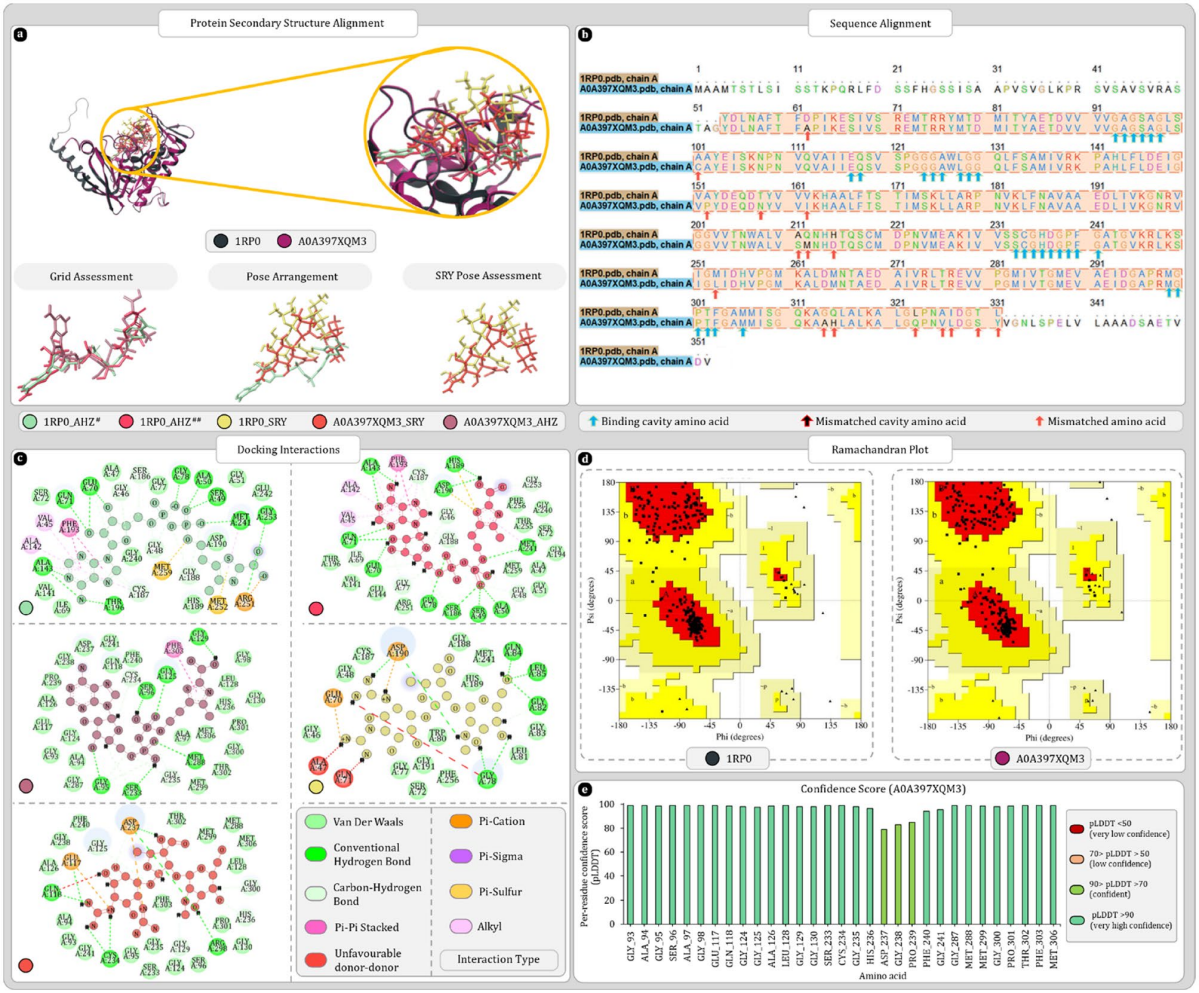
Secondary structure, sequence alignment, and docking analysis of 1RP0 and A0A397XQM3 with binding cavity analysis of A0A397XQM3 (**a**) 3D superimposition of 1RP0 and A0A397XQM3 along with 3D arrangement of ligands: Grid assessment (PDB pose of AHZ in 1RP0, redocked pose of AHZ in 1RP0, docked pose of AHZ in A0A397XQM3); Pose arrangement (PDB pose of AHZ in 1RP0, docked pose of SRY in 1RP0, docked pose of SRY in A0A397XQM3); SRY Pose arrangement (SRY in binding cavity of A0A397XQM3 and 1RP0) (**b**) sequence alignment and identification of mismatched amino acids in binding cavity of A0A397XQM3 (**c**) docking interactions of SRY and AHZ with 1RP0 and A0A397XQM3 (**d**) ramachandran plot of 1RP0 and A0A397XQM3 (**e**) per residue scores produced by AlphaFold for each residue of binding cavity.

Uniport ID A0A397XQM3 was found to be the most ideal match for the 1RP0. Results of secondary structure superimposition are given in Fig. 7a. Furthermore, sequence alignment of 278 amino acid revealed 16 mismatched amino acid and none of them were in the binding cavity Fig. 7b,c. Also, the secondary structure elements of the A0A397XQM3 protein were also maintained. Ramachandran plot analysis of 1RP0 and A0A397XQM3 respectively showed 93.2% and 93.9% amino acid in favoured region (Fig. 7d). Furthermore, the AlphaFold scores of binding cavity amino acids were in the range of highly confident and confident (Fig. 7e). Also, the molecular docking study shows that all the docked ligands were occupying the same binding cavity. The redocked AHZ ligand was also properly arranged in the binding cavity and was found to be superimposing the co-crystallized AHZ. Moreover, the pose arrangement study reveals that inside cavity docked streptomycin for both proteins was arranged similarly and the terminal rings of streptomycin were occupying the same regions as the nucleotide and thiazole rings of AHZ (Fig. 7a). Docking scores of 1RP0 with AHZ and streptomycin were respectively found to be − 16.78 and − 8.22 kcal/mol whereas for homolog A0A397XQM3, AHZ and streptomycin were giving docking score of − 11.09 and − 9.09 kcal/mol. MMGBSA of 1RP0-AHZ complex from PDB produced − 80.84 kcal/mol. After redocking score was found to be − 106.18 kcal/mol this change is expected as binding cavity was completely cleaned by removing water molecules. Here, homolog protein produced − 88.5 kcal/mol MMGBSA score with AHZ, whereas with SRY score of both 1RP0 and homolog was respectively − 53.86 and − 46.30 kcal/mol.

**Figure 8:**
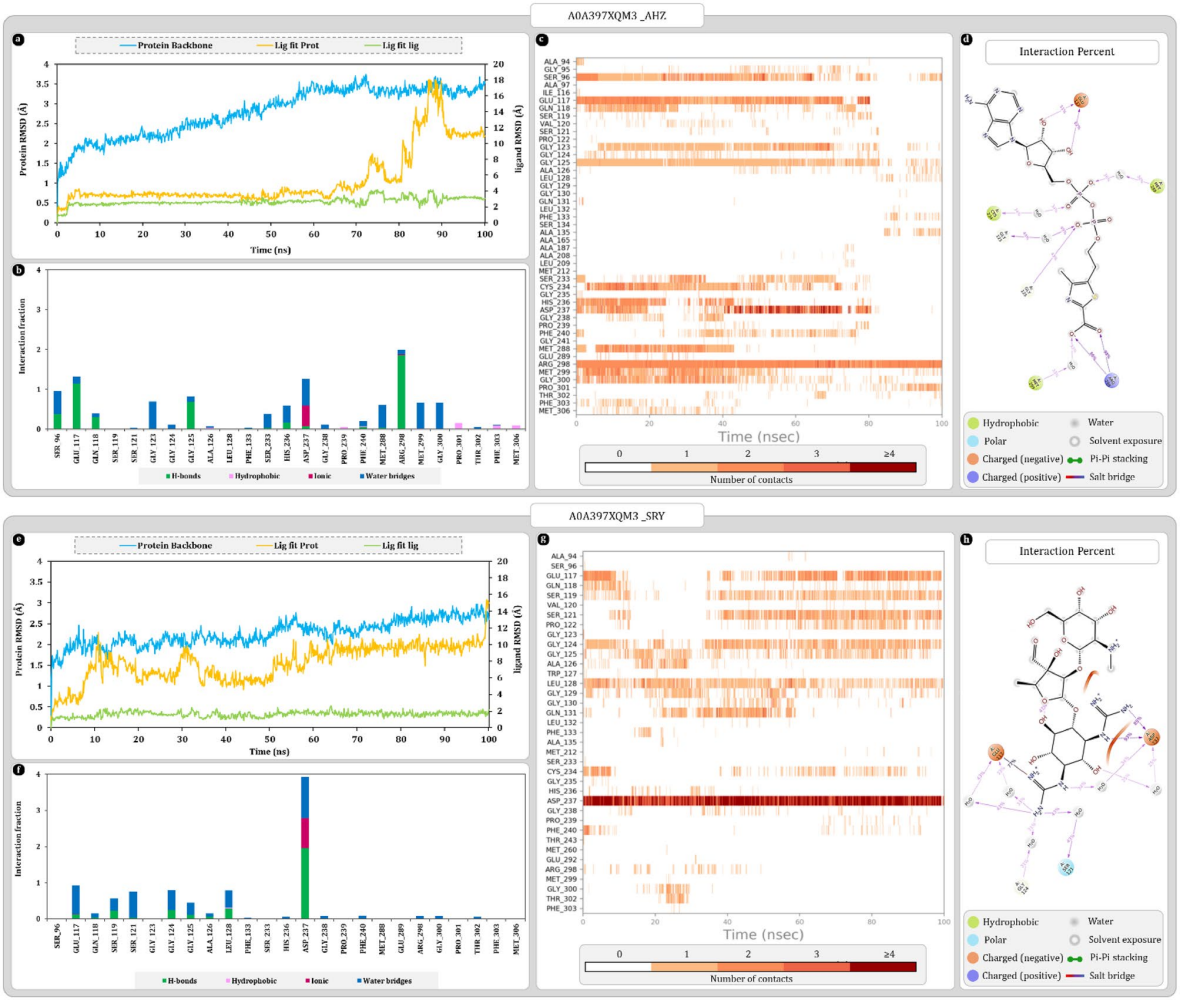
Molecular dynamics simulation trajectory analysis plots (**a**) root mean square deviation (RMSD) calculation of A0A397XQM3-AHZ complex and (**b**) interaction fraction analysis plot of A0A397XQM3 with AHZ (**c**) timeline of interactions between A0A397XQM3 and AHZ (**d**) percent interaction profile of AHZ with A0A397XQM3 (**e**) root mean square deviation (RMSD) calculation of A0A397XQM3-SRY complex (**f**) interaction fraction analysis plot of A0A397XQM3 with SRY (**g**) timeline of interactions between A0A397XQM3 and SRY (**h**) percent interaction profile of SRY with A0A397XQM3.

Protein RMSD values for A0A397XQM3-AHZ complex were gradually increasing up to 55 ns then stabilized around 3.0 ± 0.2 Å. On the other hand, ligand RMSD value “Lig Fit Prot” were stabilized around 3.7 ± 0.5 Å and remains as such for 65 ns then AHZ drastically changes pose in the cavity which gradually spikes the RMSD value to 7.5 Å at 73 ns. In 80 ns to 90 ns time ligand becomes highly active and tries to leave the cavity which spikes the RMSD, but ligand remains bound to the protein via hydrogen bonds with ARG298. Oxygens of carboxyl associated with thiazole ring forms hydrogen bonds with ARG298. After 90 ns nucleotide nitrogen base returns to the cavity which reduces the RMSD values back to 11 Å. “Lig Fit Lig” values were found to be stabilizing around 3.0 ± 0.3 Å with spike in values at 65, 73, and 80 ns which is because of internal rotations of ligand promoted by pose changes (Fig. 8a,b). Pentose moiety of nucleotide interacts with GLU117 to form hydrogen bonds that stabilizes AHZ for initial 80 ns. Protein–ligand contact timeline graph shows ARG298 as most consistently interacting amino acid throughout the simulation. Furthermore, SER96, GLU117, GLY125, CYS234, and ASP237 were the significant contributors of ligand stability for initial 60 ns. After pose changes between 80 and 90 ns ligand regains the stability via interacting with SER96, GLY123 and GLY125. Additionally, LEU128, PHE133, ALA135 and PRO301 interacts with ligand and stabilizes it again after 90 ns (Fig. 8c).

Simulation analysis of A0A397XQM3-SRY complex shows protein RMSD stabilizing in the range of 3.0 ± 0.5 Å. Ligand RMSD values were found to be increasing throughout the simulation where, initial 1–10 ns values were in the range of 4.0 ± 0.5 Å. In the range of 10–50 ns, 7.0 ± 1 Å which again increases 10.0 ± 0.5 Å for 50–95 ns. At last, after 95 ns there was a spike in the RMSD value of ligand which suggest that ligand is being propelled out of cavity. Such gradual changes in the RMSD highlights that ligand is extremely active within the cavity and complex might not remain stable for longer period of time (Fig. 8e). Protein–ligand timeline plot identifies LEU128 and ASP237 as major stability contributing amino acids. ASP237 interacts with streptidine ring sidechain and hold on to the ligand throughout the simulation (Fig. 8d,g). Percent interaction profile shows that ASP237 is forming 4 significant interactions out of which two are hydrogen bonds and other two are water-bridges with streptidine ring (Fig. 8f,h).

#### UniProt ID A0A398AN85

**Figure 9:**
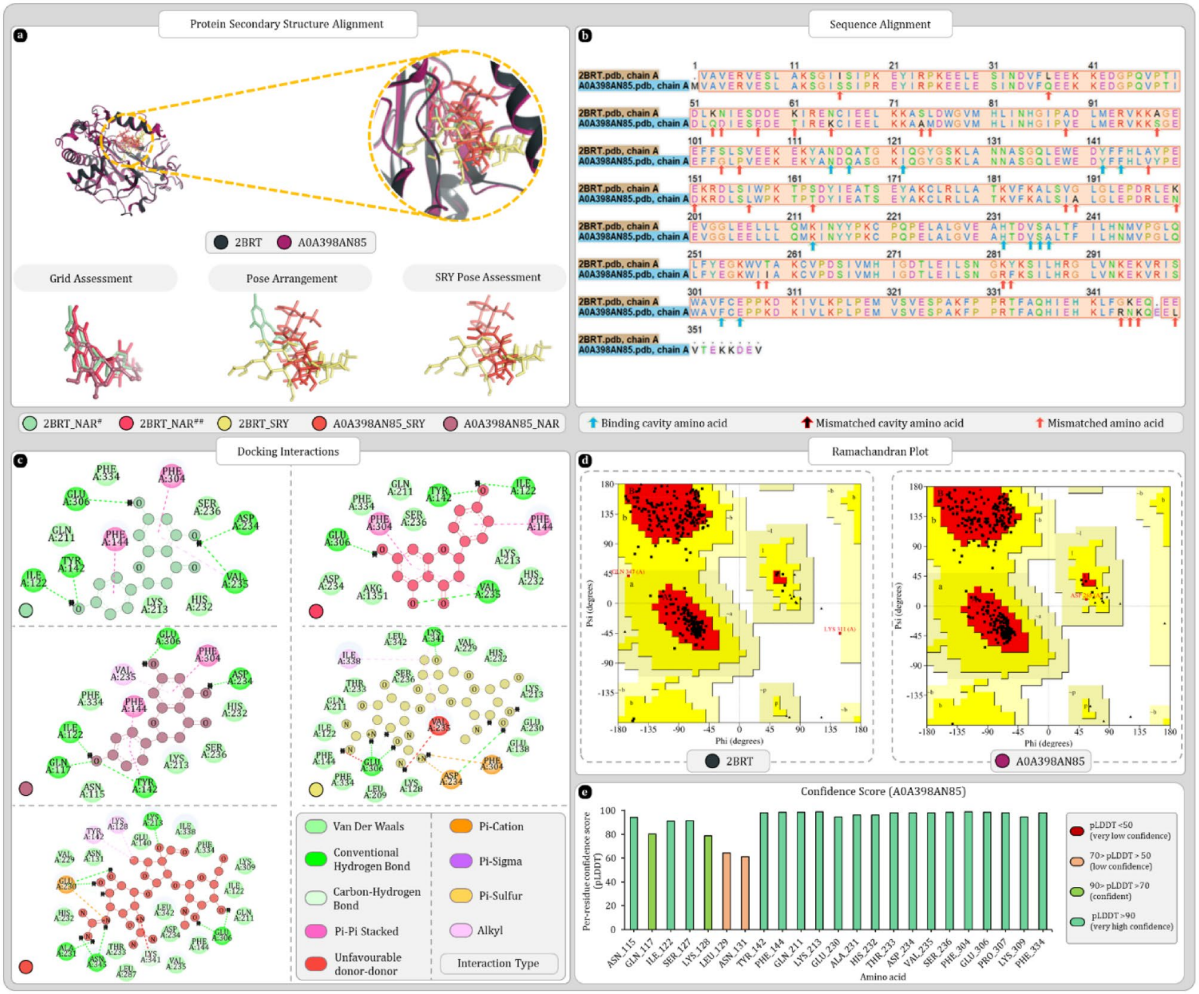
Secondary structure, sequence alignment, and docking analysis of 2BRT and A0A398AN85 with binding cavity analysis of A0A398AN85 (**a**) 3D superimposition of 2BRT and A0A398AN85 along with 3D arrangement of ligands: Grid assessment (PDB pose of NAR in 2BRT, redocked pose of NAR in 2BRT, docked pose of NAR in A0A398AN85); Pose arrangement (PDB pose of NAR in 2BRT, docked pose of SRY in 2BRT, docked pose of SRY in A0A398AN85); SRY Pose arrangement (SRY in binding cavity of A0A398AN85 and 2BRT) (**b**) sequence alignment and identification of mismatched amino acids in binding cavity of A0A398AN85 (**c**) docking interactions of SRY and NAR with 2BRT and A0A398AN85 (**d**) ramachandran plot of 2BRT and A0A398AN85 (**e**) per residue scores produced by AlphaFold for each residue of binding cavity.

BLAST analysis presents uniprot id A0A398AN85 as a best match for 2BRT. Secondary elements and sequence alignment results are given in Fig. 9a,b which shows good alignments. Sequence alignment reveals no mismatches among binding cavity amino acids. Furthermore, confidence scores were also good for most cavity amino acids except for LEU129, and ASN131 which were the only amino acids with low confidence score Fig. 9e. Ramachandran analysis yields percent scores of 91.8% and 93.7% respectively for 2BRT and A0A398AN85 which again verifies that both proteins are of good quality Fig. 9d. During docking analysis all the ligands including native ligand NAR and SRY were occupying the same binding cavity. Although, the binding pose of SRY were found to be different in 2BRT and A0A398AN85 (Fig. 9a,c). Two aromatic rings remain buried deep in the cavity whereas single ring end faces the mouth of cavity for NAR whereas streptidine ring of SRY was arranged in the deep end of cavity and L-glucosamine ring was facing the mouth of cavity. Docking score of 2BRT with NAR and SRY was − 6.166 and − 9.393 kcal/mol. Whereas A0A398AN85 was giving the docking score of − 6.126 and − 8.592 kcal/mol respectively with NAR and SRY. In both cases, SRY produced batter binding scores with both proteins (Fig. 9c).

**Figure 10:**
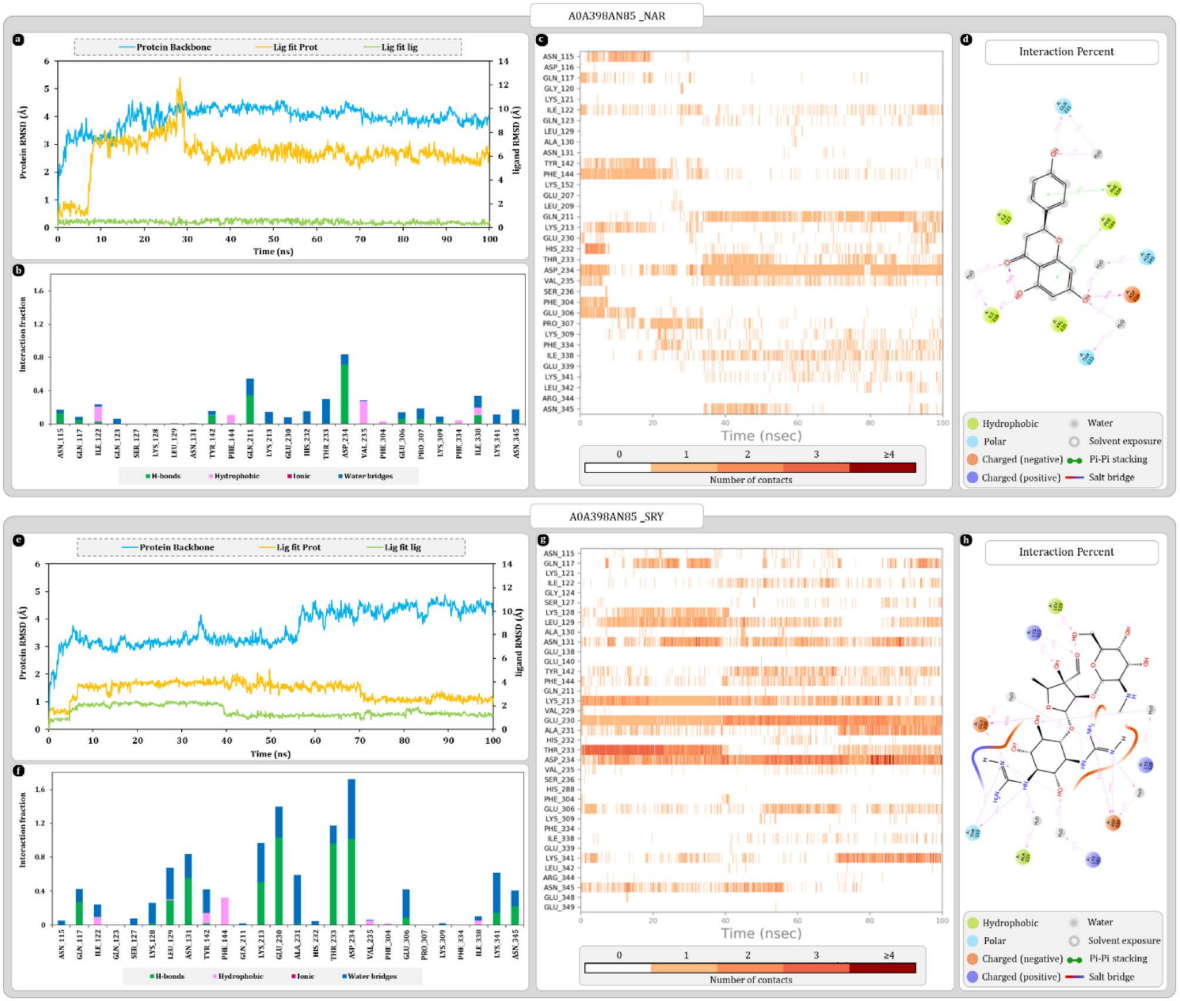
Molecular dynamics simulation trajectory analysis plots (**a**) root mean square deviation (RMSD) calculation of A0A398AN85-NAR complex and (**b**) interaction fraction analysis plot of A0A398AN85 with NAR (**c**) timeline of interactions between A0A398AN85 and NAR (**d**) percent interaction profile of NAR with A0A398AN85 (**e**) root mean square deviation (RMSD) calculation of A0A398AN85-SRY complex (**f**) interaction fraction analysis plot of A0A398AN85 with SRY (**g**) timeline of interactions between A0A398AN85 and SRY (**h**) percent interaction profile of SRY with A0A398AN85.

MD simulation analysis of A0A398AN85-NAR complex was producing the RMSD values for protein backbone in the range of 4.0 ± 0.5 Å. Here, lig fit lig remains stable at 1.0 ± 0.2 Å as internal rotations of ligand are bare minimum due to small size and only one major rotatable bond. Whereas NAR pose was settling with RMSD of 6.0 ± 0.3 Å which suggests major changes in pose arrangement compared to docked pose regardless of these changes’ ligand is still within the cavity. First pose change occurs at 10 ns here, ligand shifts in the cavity and spike of 8 Å in ligand RMD is registered. Later around 29 ns ligand shifts back closer to original pose which results in reduction of RMSD by 2 Å (Fig. 10a). For initial 10 ns NAR was forming hydrogen bonds with GLU306 and ASP234 after stabilization around 30 ns ASP234 interacts with ligand again (Fig. 10b–d). Furthermore, GLN211 alongside ASP234 contributes significantly to stabilizing NAR. VAL235 and IEL338 are the other contributors that forms hydrophobic interactions with NAR. Here, it is also noteworthy that IEL122, and PHE144 also forms hydrophobic interactions with NAR but only during the phase of higher RMSD at around 0 to 20 ns phase of simulation (Fig. 9b–d).

On the other hand, simulation of protein with SRY was producing the RMSD values of 4.0 ± 0.5 Å which acceptable variation. Ligand RMSD values were found to be stabilizing around 3.0 ± 0.5 Å within first 10 ns and then only slight decrease of 1 Å was noted around 68 to 70 ns timeframe. At around 40 ns l-glucosamine ring breaks the hydrogen bond with LEU129 and rotates to be free in the cavity for a while then at 69 ns it starts periodic interactions with GLU306 simultaneously streptose ring also interacts with LYS213 which stabilizes the ligand RMSD and brings it down by 1 Å (Fig. 10e,f). Interaction fraction profile reveals that most stable hydrogen bonds were being formed by ASN131, LYS213, GLU230, THR233 and ASP234. LYS213 was actively engaging with streptose ring whereas rest of the amino acids were interacting with streptidine ring (Fig. 10f–h). Strongest hydrophobic interaction was formed by PHE144, and it was having periodic presence throughout the simulation. Among other significant interactions water bridges were also present in most amino acids, including ASN131, GLU230, ALA231, and ASP234 (Fig. 9f).

#### UniProt ID A0A397XTZ2

**Figure 11:**
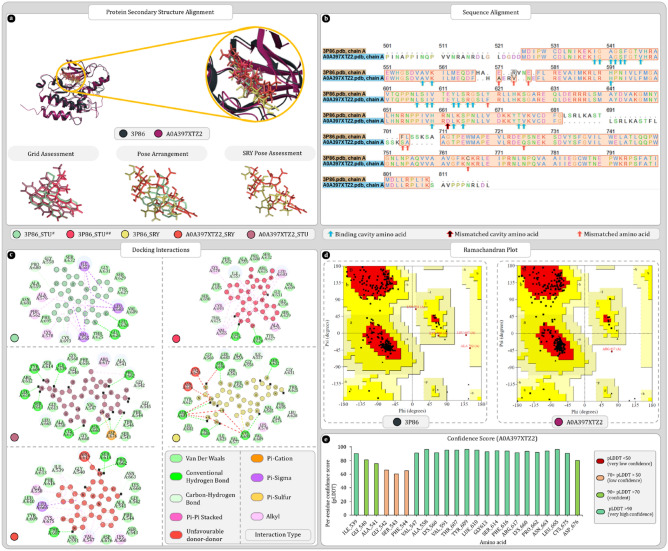
Secondary structure, sequence alignment, and docking analysis of 3P86 and A0A397XTZ2 with binding cavity analysis of A0A397XTZ2 (**a**) 3D superimposition of 3P86 and A0A397XTZ2 along with 3D arrangement of ligands: Grid assessment (PDB pose of STU in 3P86, redocked pose of STU in 3P86, docked pose of STU in A0A397XTZ2); Pose arrangement (PDB pose of STU in 3P86, docked pose of SRY in 3P86, docked pose of SRY in A0A397XTZ2); SRY Pose arrangement (SRY in binding cavity of A0A397XTZ2 and 3P86) (**b**) sequence alignment and identification of mismatched amino acids in binding cavity of A0A397XTZ2 (**c**) docking interactions of SRY and STU with 3P86 and A0A397XTZ2 (**d**) ramachandran plot of 3P86 and A0A397XTZ2 (**e**) per residue scores produced by AlphaFold for each residue of binding cavity.

BLAST analysis revealed A0A397XTZ2 as homolog of 3P86. Secondary structure alignment and sequence alignment results are given in Fig. 11a,b. A0A397XTZ2 shares same binding cavity as 3P86 for ligand STU furthermore, the alpha and beta sheets of homolog are also conserved. The Ramachandran plots of 3P86 and A0A397XTZ2 respectively provides 86.8% and 87% amino acids in most favoured regions which is below threshold of 90%. Hence, both proteins were moderate in their quality (Fig. 11d). Furthermore, docking analysis positioned the ligands in same binding cavity and the scores produced by 3P86 with STU and SRY are − 9.29 kcal/mol and − 11.171 kcal/mol. Whereas, A0A397XTZ2 produced − 6.11 kcal/mol and − 8.80 kcal/mol for STU and SRY. In case of SRY, binding cavities of both proteins were giving unfavourable donor-donor interactions (Fig. 11c). In confidence score analysis, most amino acids were either in highly confident or confident zone, except for three amino acids GLY542, SER543, and PHE544 that were having low confidence (Fig. 11e).

**Figure 12:**
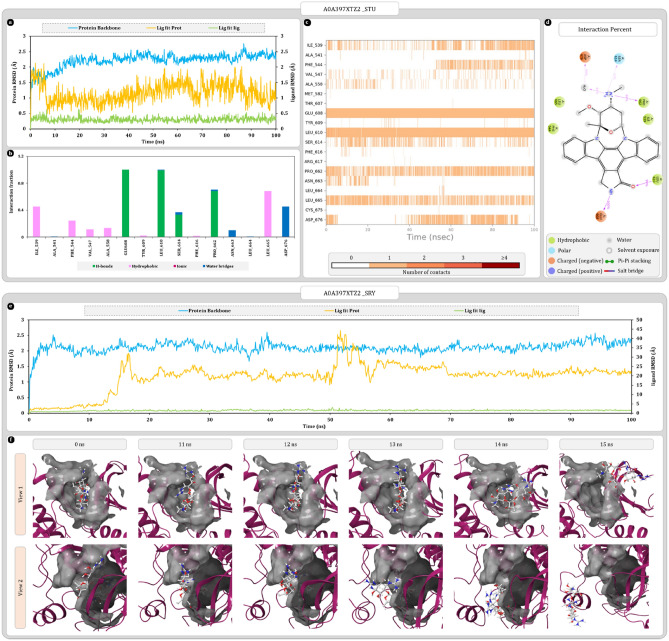
Molecular dynamics simulation trajectory analysis plots (**a**) root mean square deviation (RMSD) calculation of A0A397XTZ2-STU complex and (**b**) interaction fraction analysis plot of A0A397XTZ2 with STU (**c**) timeline of interactions between A0A397XTZ2 and STU (**d**) percent interaction profile of STU with A0A397XTZ2 (**e**) root mean square deviation (RMSD) calculation of A0A397XTZ2-SRY complex (**f**) movement of SRY in binding cavity of A0A397XTZ2 at 0 ns, and 11–15 ns from two different angles.

In MD simulation analysis A0A397XTZ2-STU complex was found to be stable with RMSD value of for protein backbone 2.3 ± 0.3 Å. Similarly, RMSD values for ligand were also minimal, 1.0 ± 0.5 Å. Here, amino acid GLU608 and LEU610 were forming hydrogen bonds and contributing consistently to ligand stability during the simulation. Furthermore, ILE539, PHE544, and LEU665 were making hydrophobic interactions to stabilize the ligand in binding cavity (Fig. 12a–d). Although similar stability was not observed for A0A397XTZ2-SRY complex. Here, protein backbone RMSD values similar to A0A397XTZ2-STU complex, 2.3 ± 0.3 Å. On the other hand, ligand RMSD values, were showing the spike of 22 Å at 14 ns suggesting the breakage of complex and escape of ligand from binding cavity (Fig. 12e,f).

**Figure 13:**
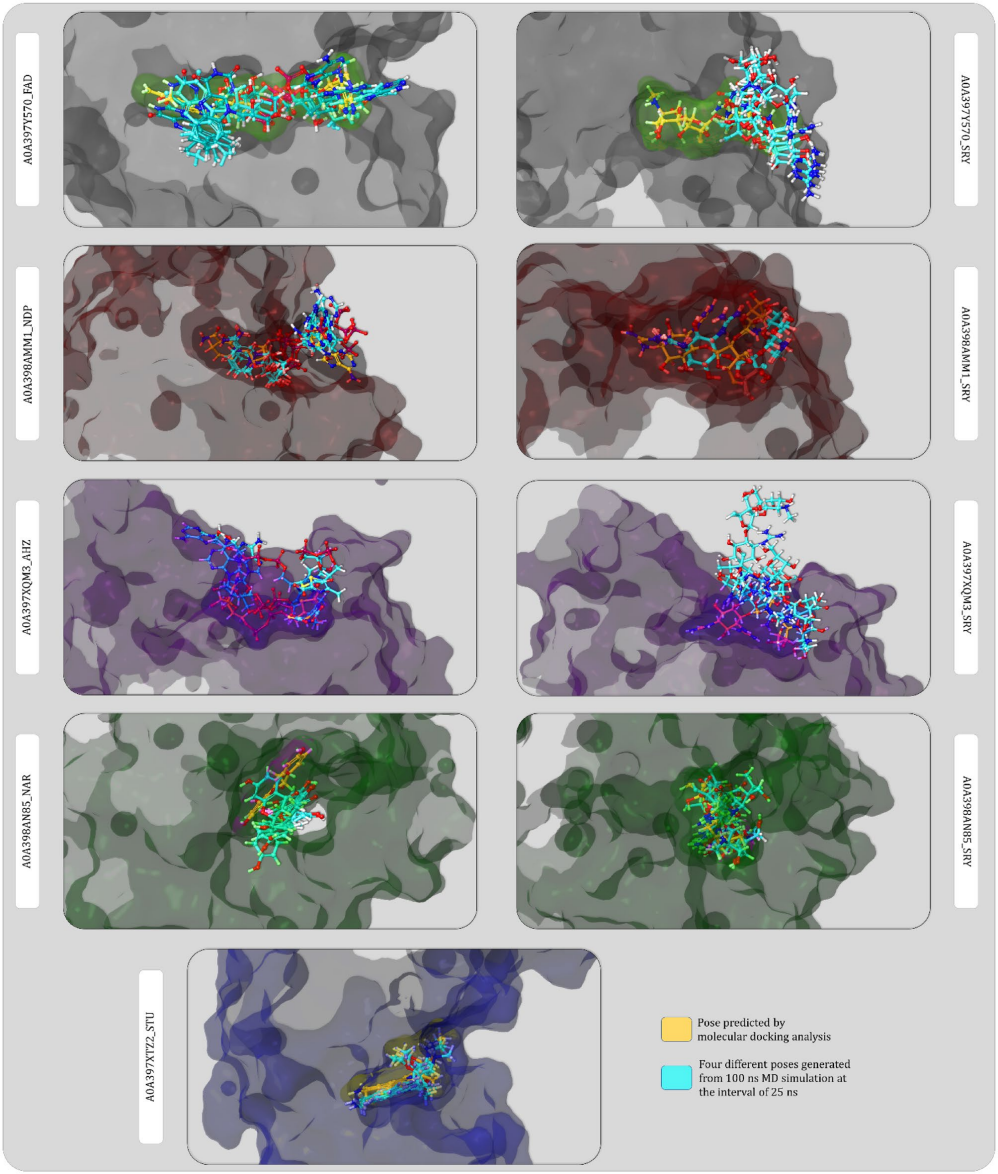
Protein ligand interaction poses of MD simulation extracted from simulation data at the interval of 25 ns. Here, protein surfaces and ligand surfaces were produced at frame 0 with docked posed hence, they can be used to get the idea of ligands movement away from initial docked pose (yellow) compared pose at various interval (bright blue).

Figure 13 represents the poses of ligand and proteins extracted from the MD simulation data at the interval of 25 ns. The video output of all the simulations performed are provided as Supplementary Material files (S1).

## Discussion

SRY is aminoglycoside antibiotic that was once being researched for its ability to combat biotic stress and support plant growth, but studies revealed the negative effects on plant physiology. Many speculations predicting the molecular functions of SRY in plants are there but scientific evidence is still lacking^15,19^. In current study, molecular docking, MM-GBSA and MD simulation tools were used to analyse the molecular interactions of streptomycin within young seedling plants of *Brassica napus*. Among the targets there were three proteins that were giving stable binding complex with streptomycin namely they were acyl-CoA oxidases (2IX5; A0A397Y570), protochlorophyllide reductase B (7JK9; A0A398AMM1) and leucoanthocyanidin dioxygenase (2BRT; A0A398AN85). Here, protochlorophyllide reductase was giving the most stable complex with least number of fluctuations. Among remaining thiamine thiazole synthase (1RP0; A0A397XQM3) was providing a relatively stable complex but here only single amino acid ASP237 was holding on to the ligand at the end of simulation which suggests that complex might not remain stable for long period of time. Another lead Serine/threonine-protein kinase CTR1 (3P86; A0A397XTZ2) rejected the streptomycin immediately and the simulation of docked complex was broken withing 15 ns of simulation and ligand never returned in the cavity during entire simulation.

Acyl-CoA oxidases protein is required in plants for fatty acid breakdown through peroxisomal β-oxidation^32^. In plants this enzyme is found in different subcellular locations including cytosol, glyoxysome, and peroxisome where it has been associated with range process such as embryo development, postembryonic development, and fatty acid β-oxidation^33,34^. During early developmental stages the enzyme is found abundantly in etiolated cotyledons. Furthermore, evidence suggest that ACX induction occurs when plant is facing dehydration, and high abscisic acid concentrations^35,36^. Additionally, among six different isozymes ACX1, has been linked to the synthesis of the jasmonate which is associated with biotic and abiotic stress management in plants^37^. Here, streptose in streptomycin and ribose in adenosine monophosphate of FAD shares similarity of sharing sugar origin. Although, there are no studies currently available that has assessed the effects of streptomycin on plant lipid synthesis profile. Study on chloroplast containing *Euglena gracilis* reports changes in galactolipid composition between groups exposed and unexposed to streptomycin.

Protochlorophyllide reductase B is actively expressed in etiolated seedlings and adult plants. This enzyme is required for phototransformation of protochlorophyllide (Pchlide) to chlorophyllide (Chlide) which is required for photosynthesis in plants^38–40^. Furthermore, multiple studies provide evidence that SRY affects the chlorophyll formation abilities of plants. Furthermore, barley, rye, carrot tumour tissue, pine seedlings, Chlorella, cress, and radish exposed to SRY were showing leaf yellowing due to chlorosis. Similarly, *Euglena gracilis var. bacillar* subjected to 100 ppm of streptomycin was showing permanent bleaching of^13^. Hence, it is indisputable that SRY does have direct impact on chlorophyll synthesis process of plants. In current study there were apparent changes in the colour of plant where seeds exposed to streptomycin were pale yellow in colour. Further in silico analysis is also supporting this information as the complex of protein with SRY was most stable among all tested proteins suggesting strong affinity between protein and SRY. This protein is mostly reported in photosynthetic tissue of plants such as flowers, upper leaves, rosette and cauline leaves, stem. Also, the protein is absent in non-photosynthetic tissues such as roots and seeds^41^.

Leucoanthocyanidin dioxygenase belongs iron/ascorbate-dependent oxidoreductase family of enzymes. In plants, it is required for synthesis of anthocyanin and protoanthocyanidin through oxidation of leucoanthocyanidins into anthocyanidins. Furthermore, in vitro assay has produced evidence suggesting that enzyme has flavonol synthase activity where dihydrokaempferol and dihydroquercetin are used as a substrate to produce kaempferol and quercetin, respectively^42–45^. Disruption studies shows the absence anthocyanin alongside accumulation of protoanthocyanidin intermediates with small vacuoles in leaf epidermal cells. Also, it is noteworthy that absence of enzyme is not lethal enough to kill the plant but disrupts the operational pathway that is necessary for growth, hence, retards the growth as it is frequently observed in many studies involving SRY^38,46,47^. Experimental evidence has proved the presence of enzyme in young seedlings of *Arabidopsis thaliana*, where the light, sucrose and methyl jasmonate are established as an inducer of the pathway necessitating the presence of leucoanthocyanidin dioxygenase^47–49^.

Here, thiamine thiazole synthase and serine/threonine-protein kinase CTR1 did manage to produce decent scores on docking and MM-GBSA but MD simulation study results does not favour them as a potential target for SRY. In conclusion, study represents acyl-CoA oxidases, protochlorophyllide reductase B and leucoanthocyanidin dioxygenase as potential targets for SRY. Also, the intention here is to provide clear targets for experimental study to confirm the intracellular activities of SRY.

### Supplementary Information

Supplementary Videos.

Supplementary Video 1.

Supplementary Video 2.

Supplementary Video 3.

Supplementary Video 4.

Supplementary Video 5.

Supplementary Video 6.

Supplementary Video 7.

Supplementary Video 8.

Supplementary Video 9.

Supplementary Video 10.

Supplementary Video 11.

Supplementary Video 12.

Supplementary Video 13.

Supplementary Video 14.

Supplementary Video 15.

## Data Availability

All the relevant data is contained within the manuscript. Additional raw data will be available upon request to the corresponding author.
