# Supplementary material for: Probing the effects of streptomycin on Brassica napus germination and assessing its molecular interactions using extensive molecular dynamics (MD) simulations
Source: Sci Rep. 2023 Nov 4;13:19066. doi: 10.1038/s41598-023-46100-4 (PMC10625591; doi:10.1038/s41598-023-46100-4)
Supplement: Supplementary file 1 — Supplementary Videos. [file 41598_2023_46100_MOESM1_ESM.docx]

Video_1. MD simulation of A0A397Y570_FAD complex representing the ligand movement in cavity from the reference frame 0 highlighted through surface gray.

Video_2. MD simulation of A0A397Y570_FAD complex representing the hydrogen bond forming amino acids.

Video_3. MD simulation of A0A397Y570_FAD complex representing the hydrophobic interactions.

Video_4. MD simulation of A0A397Y570_SRY complex representing the hydrogen bonds.

Video_5. MD simulation of A0A397Y570_SRY complex representing the hydrophobic interactions.

Video_6. MD simulation of A0A398AMM1_NDP complex representing the hydrogen bonds.

Video_7. MD simulation of A0A398AMM1_SRY complex representing the hydrogen bonds.

Video_8. MD simulation of A0A397Y570_SRY complex representing the ligand movement in the cavity from the reference frame 0.

Video_9. MD simulation of A0A397XQM3_AHZ complex representing the hydrogen bonds.

Video_10. MD simulation of A0A397XQM3_AHZ complex representing the movement of ligand in cavity.

Video_11. MD simulation of A0A397XQM3_SRY complex representing hydrogen bonds.

Video_12. MD simulation of A0A398AN85_NAR complex representing hydrogen bonds.

Video_13. MD simulation of A0A398AN85_SRY complex representing hydrogen bonds.

Video_14. MD simulation of A0A397XTZ2_STU complex representing hydrogen bonds.

Video_15. MD simulation of A0A397XTZ2_SRY complex representing the escape of ligand from binding cavity.
